# Lost Narratives: Identifying Predictors of Attrition and Differences in Recruitment Effort in a Longitudinal Study on Child Maltreatment

**DOI:** 10.1177/10775595251352425

**Published:** 2025-07-04

**Authors:** Jan Keil, Josephine Breuer, Romy Küchler, Angelika J. Bracher, Charlotte C. Schulz, Dorukhan Açıl, Sarah Bergmann, Nina Alexander, Tobias Stalder, Robert Miller, Maria Licata-Dandel, Volker Mall, Michaela Augustin, Anne Sophie Wenzel, Georg von Polier, Daniel Radeloff, Kai von Klitzing, Lars O. White

**Affiliations:** 1 Department of Child and Adolescent Psychiatry, Medical Faculty, 39066 Leipzig University, Leipzig, Germany; 2 27184 Max Planck Institute for Human Cognitive and Brain Sciences, Leipzig, Germany; 3 International Psychoanalytic University Berlin, Berlin, Germany; 4 Department of Psychiatry and Psychotherapy, 9377 University of Marburg, Marburg, Germany; 5 Center for Mind, Brain and Behavior, 9377 University of Marburg, Marburg, Germany; 6 Department for Clinical Psychology and Psychotherapy, 14312 University of Siegen, Siegen, Germany; 7 Department of Psychological Methods, 530999 Psychologische Hochschule Berlin, Berlin, Germany; 8 Department of Psychology, Charlotte-Fresenius-University, Munich, Germany; 9 Social Pediatrics, 155892 TUM School of Medicine, Technical University of Munich, Munich, Germany; 10 38180 kbo-Kinderzentrum, Munich, Germany; 11 German Center for Child and Adolescent Health (DZKJ), Partner Site Munich, Germany; 12 Department of Clinical Child and Adolescent Psychology and Psychotherapy, University of Bremen, Bremen, Germany

**Keywords:** longitudinal study, maltreatment, attrition, re-assessment effort

## Abstract

Longitudinal research on the adverse consequences of childhood maltreatment has recently gained significant traction. However, systematic attrition, partly due to specific subsample recruitment needs, threatens the validity of this research. Furthermore, studies specifically analyzing these factors in the field of maltreatment research remain scarce. We utilized data from a longitudinal study comprising *N* = 863 participants (*M_Age_* = 10.23 years, 47.5% female) and their caregivers at T1, and 616 participants (*M_Age_* = 17.95 years, 50.0% female) at T2. We determined the attrition rate and analyzed psychosocial and socioeconomic predictors of attrition. Additionally, we examined differences in re-assessment efforts between maltreated and non-maltreated youth for T2. Findings indicate a comparatively low attrition rate of 28.6% over nearly 8 years. Participants’ maltreatment experiences, externalizing symptoms, and lower household income predicted higher attrition risk, while interim subsample study participation reduced this risk. Maltreatment experiences, lower household income, and higher age were also associated with increased re-assessment effort. Our study provides insights into predictors of systematic attrition in a longitudinal study with maltreated and non-maltreated youth. It highlights the need for tailored retention strategies, frequent contact with families, and targeted resource allocation to mitigate systematic attrition.

## Introduction

Maltreatment ranks among the most detrimental factors affecting the well-being of children and adolescents. Numerous studies consistently link childhood maltreatment to an increased risk of poor mental health outcomes across the lifespan (e.g., Li et al., 2016). The importance of longitudinal research in this area cannot be overstated, as it is crucial for determining the extent to which maltreatment temporally precedes poor developmental outcomes – a key prerequisite for establishing causality in a field where experimental designs are not feasible. However, longitudinal research involving high-risk populations often faces significant challenges, with systematic attrition among the most prominent (Ellard-Gray et al., 2015). Study samples affected by maltreatment frequently encounter numerous psychosocial difficulties, making them especially difficult to recruit and retain for longitudinal assessments (Kinard, 2001; Kisely et al., 2020). These challenges may result in selective or systematic attrition, whereby individuals at the highest risk are more likely to drop out, thereby threatening the validity of longitudinal studies in this field (Barry, 2005; Feldstein Ewing et al., 2022; Loeber & Farrington, 1994). Despite a growing body of literature on the determinants of attrition and retention strategies for “hard-to-reach” or “hidden” populations (Dyson et al., 2021), systematic analyses of these factors in maltreatment research involving children and adolescents remain scarce. This study aims to address this critical gap by providing data from a longitudinal cohort study. By including both maltreated and non-maltreated youth, it seeks to identify factors that could mitigate systematic attrition bias, thereby enhancing the generalizability of findings and providing more reliable insights to inform interventions.

### Attrition Rates

Attrition (i.e., participant loss to follow-up) has been a topic of concern across various research fields, particularly in studies involving both high- and low-risk youth and adult samples. Longitudinal studies with underage participants show considerable variability in attrition rates (Rothman, 2009). For instance, Dimakos et al. (2023) reported an attrition rate of 58.5% for reassessments conducted after 10 years, while Delfabbro et al. (2017) found that 40% of their annually assessed sample dropped out after the first survey, resulting in an overall attrition rate of 81.83% over 10 years. In studies involving maltreated and non-maltreated children, annual attrition rates also vary widely, ranging from 7% (Cicchetti & Manly, 1990) to 40% (Valentino et al., 2011). A recent 10-year longitudinal study with *N* = 454 maltreated and non-maltreated participants reported an overall attrition rate of 22%, which Negriff et al. (2020) considered relatively low given the high-risk nature of the sample. These rates are also comparable to those reported in a large 40-year longitudinal birth cohort study including both maltreated and non-maltreated participants, which found attrition rates of approximately 27% after 5 and 14 years (Kisely et al., 2020). Additionally, a review of obesity prevention and treatment trials involving minority or low-income youth samples (Cui et al., 2015) highlighted that studies with shorter reassessment intervals - such as one year - tend to have lower attrition rates (12.8%). This trend was even more pronounced in studies focusing exclusively on children (9.8%) or targeting low-risk samples (10%). In contrast, studies spanning more than one year reported higher attrition rates (26.0%), including those involving both children and parents (14.4%) or recruiting obese children (20.4%). Overall, these findings suggest that attrition rates are highly variable and strongly influenced by study design and sample characteristics.

### Determinants of Attrition

Several studies have sought to identify psychosocial factors in population and clinical samples of children and adolescents that may contribute to study attrition. Some research has found an association between the presence of a psychiatric condition and an increased risk of attrition (Williams et al., 2013). However, more fine-grained analyses of the relationship between externalizing and internalizing symptoms and attrition have yielded mixed results. While some research reports that externalizing - but not internalizing - problems are linked to premature treatment dropout in maltreated children (Wamser-Nanney & Steinzor, 2017), other findings suggest that externalizing and internalizing symptoms increase the likelihood of attrition among maltreated children (McNamara, 2001). Furthermore, a recent study found that moderate to high levels of externalizing symptoms were specifically associated with attrition, whereas internalizing symptoms showed a curvilinear relationship, with both very low and very high levels predicting higher treatment dropout rates in maltreated children (Tebbett et al., 2018). In contrast, several longitudinal studies with community samples have found only weak or no associations between the number of psychological symptoms (Allehoff et al., 1988) or general psychological well-being (Delfabbro et al., 2017) and study attrition. Additionally, findings from a parenting intervention study suggest that higher problematic parenting scores, greater parenting stress, and lower levels of social support may predict attrition (McWey et al., 2015).

Socioeconomic status (SES) has also been examined as a potential predictor of attrition. Some studies indicate that socioeconomic disadvantage (Kazdin et al., 1993), lower caregiver education, and lower annual income (Williams et al., 2013) are associated with higher attrition rates. In contrast, Allehoff et al. (1988) did not find a relationship between attrition and various SES indicators such as social class or living space. Interestingly, findings from child psychotherapy studies suggest a phase-specific association between SES and attrition, with some evidence indicating that lower SES may be linked to elevated levels of attrition in early study phases, while higher SES could be associated with attrition in later phases (Armbruster & Kazdin, 1994). In line with this, it has been suggested that attrition during specific treatment phases may be influenced by factors such as minority status and the severity of depression symptoms (Gonzalez et al., 2011). These complex interactions could potentially explain the inconsistencies observed in previous findings.

Age and gender have also been examined as potential predictors of attrition. Evidence suggests that participants approaching the legal age of adulthood are more likely to drop out than both older adults (Eaton et al., 1992; Vanable et al., 2002) and school-aged children (Delfabbro et al., 2017). It has been argued that, as adolescents grow older and near graduation, they are more likely to begin paid employment, pursue further education, or move out of their parental homes - factors that can complicate researchers’ efforts to maintain contact and follow up (Delfabbro et al., 2017). With regard to gender, findings have been relatively consistent, indicating that boys are more likely to drop out than girls (Delfabbro et al., 2017; Eaton et al., 1992; Kisely et al., 2020). However, a comprehensive explanation for these gender differences has yet to be established.

In summary, the high variability in findings regarding psychosocial and socioeconomic factors suggests that their relationship with attrition may be highly context-dependent, influenced by specific population characteristics and study designs. Although longitudinal cohort and intervention studies involving at-risk populations provide valuable insights, research systematically analyzing the determinants of attrition in studies involving both maltreated and non-maltreated youth remains scarce. To our knowledge, no study has comprehensively investigated these factors within this specific context.

### Differences in Re-Assessment Effort

In addition to investigating potential determinants of study attrition, several studies have explored strategies to mitigate attrition rates. In this context, numerous literature reviews (e.g., Bonevski et al., 2014) and meta-analyses (e.g., Brueton et al., 2013; Cui et al., 2015; Lindsey et al., 2014) have focused on identifying effective recruitment and retention strategies for hard-to-reach populations. These reviews have highlighted a range of strategies that may help maintain participant involvement, including, but not limited to, comprehensive tracking procedures, incentives, and frequent participant contact (Dyson et al., 2021). However, results regarding the effectiveness of these strategies have been somewhat inconsistent, suggesting that approaches must be tailored to the specific needs of the target population (Bonevski et al., 2014). This is particularly important given that many strategies for vulnerable populations are resource-intensive.

Surprisingly, despite numerous studies on the determinants of attrition and retention strategies, few have specifically examined differences in re-assessment efforts between vulnerable populations and control groups, especially in maltreatment research. As one of the few studies in this area, Dyson et al. (2021) reported an average recruitment time of 5 hours for domestically violent fathers and controls participating in a two-wave longitudinal study. The authors found no significant differences between the groups in terms of missed or rescheduled appointments or re-assessment efforts for the first appointment. However, more effort was required for domestically violent fathers to re-attend the second appointment. These findings suggest that resource-intensive recruitment strategies may be particularly beneficial for certain subgroups or study phases. A more nuanced understanding of recruitment differences could optimize resource allocation, potentially reducing overall research costs and alleviating a significant barrier for researchers and funding organizations.

### The Current Study

The present study aims to address the research gaps outlined above, using data from the AMIS- project (White et al., 2015). AMIS is a large-scale longitudinal investigation designed to explore developmental trajectories linking childhood maltreatment with psychiatric symptoms and disorders, focusing on the interaction of maltreatment characteristics with cognitive-emotional and social processes, as well as endocrine, metabolomic, and (epi-) genomic patterns.

Specifically, this study had two primary objectives: First, we aimed to analyze psychosocial and socioeconomic determinants associated with study attrition between the first (T1) and second (T2) data collection waves in both maltreated and non-maltreated youth. Based on existing literature, we hypothesized that higher levels of participant and caregiver maltreatment experiences and psychopathology, as well as lower SES, would be associated with a higher likelihood of study attrition. We also anticipated that older and male participants would exhibit a greater likelihood of attrition. Furthermore, we examined whether different forms of maltreatment exposure (i.e., abuse, neglect, and emotional maltreatment) differentially predict study attrition and whether the breadth of exposure, defined by the number of experienced subtypes, was associated with attrition. Second, we sought to examine differences in re-assessment efforts at T2 between the maltreated and non-maltreated subsamples. Based on prior research, we hypothesized that re-assessment efforts would be greater for maltreated than for non-maltreated participants.

## Method

### Participants

The initial sample of 966 families in the AMIS-study was recruited from two German cities, Leipzig and Munich. Due to changes in participating research facilities, we were only able to approach *N* = 863 (*M*_Age_ = 10.23, *SD* = 3.17, 47.5% female) families for re-assessment, all of whom had participated in the first wave of data collection (T1) between 2012 and 2015 in Leipzig. Leipzig is an eastern German city with a population of approximately 600,000 people and above-average poverty rates. Recruitment sources for T1 included child protection services (CPS; *n* = 119, 98.3% maltreated), child and adolescent psychiatric services (CAPS; *n* = 169; 46.7% maltreated), and community outlets, including daycare centers, general practitioners, and the resident registration office (*n* = 575; 21.0% maltreated) in Leipzig. To include preschool and early school-aged children at psychiatric risk, a subgroup of community children was intentionally oversampled for elevated internalizing symptoms exceeding the borderline cut-off on the Strengths and Difficulties Questionnaire (SDQ; R. Goodman, 1997; Klitzing et al., 2012). For T1, we detected maltreatment histories in *n* = 317 (36.7%) of these children and adolescents. In the second wave of data collection (T2) between 2019 and 2023, *n* = 616 individuals (*M*_Age_ *=* 17.95, *SD* = 3.13, 50.0% female) were reassessed. The mean duration between both waves was 7.84 years (*SD* = 0.84). Of these participants, *n* = 314 (52.4%) had known histories of maltreatment. Maltreatment histories were only identified during the second data collection wave (i.e., prospectively) for *n* = 131 (41.7%) individuals (see Table 1 for the sociodemographic characteristics of the sample at T1 and T2).

**Table 1.** Sociodemographic Characteristics of the AMIS-Sample During the First (T1) and the Second (T2) Wave of Data Collection.

	Sample T1 (*N* = 863)	(Sub-) sample T2 (*n* = 616)
Age of participants (*M, SD*)	10.23 (2.37)	17.95 (3.13)
Female gender (*n,* %)	410 (47.5)	308 (50.0)
Net-income bracket (Median)	2000€–2500€	2500–3000 €
Caregiver school education (Median)	High school diploma	High school diploma
Presence of maltreatment experiences (*n*; %)	317 (36.7)	131 (41.7)

### Procedure

During each wave, families attended one or two appointments, each lasting approximately 3 hours. The majority of instruments (i.e., interviews, questionnaires, and experimental tasks), including the maltreatment assessment, were administered to participants and primary caregivers in both waves. Additionally, we administered questionnaires to the second caregiver and a teacher, with participant and caregiver consent, and reimbursed caregivers or adult participants. Underage participants were compensated with a gift in both waves. In addition to the two data collection waves of the main study, we conducted two interim assessments in Leipzig. These are considered interim assessments rather than data collection waves, because they differed significantly from the main study in terms of methodology and research goals. Interim 1 (2017–2018, *n* = 636) focused on low-threshold psychopathological monitoring of the sample, relying solely on (screening) questionnaires completed by participants, caregivers, or both. Importantly, it did not include key measures of maltreatment exposure, interviews, experimental tasks, or the collection of biological samples. Interim 2 (2018–2020, *n* = 110) targeted the specific research question on the neuropsychological effects of maltreatment experiences in a carefully matched, selective subsample (see Schulz et al., 2022; for more information). An overview of the data collection waves, interim assessments, and corresponding (sub-) samples is provided in Table S1.

It is important to note that data collection at the second time point was suspended for several months due to the COVID-19 pandemic. Ethical approval for the study was granted by the university’s Institutional Review Board. Caregivers and youth provided informed oral and written consent before participation.

### Measures

#### Maltreatment Experiences

The Maltreatment Classification System (MCS; Barnett et al., 1993) was used to assess participants’ maltreatment experiences. The MCS represents a valid, reliable coding system with semantic definitions and examples of six maltreatment subtypes (Failure to Provide, Lack of Supervision, Sexual Abuse, Physical Abuse, Emotional Maltreatment, Moral-legal/Educational Maltreatment; Manly et al., 2013). The AMIS- group was trained and supervised throughout both waves by an MCS author (Jody T. Manly, PhD) to ensure high quality and international comparability of ratings. Reports of potential maltreatment incidents came from two sources: For CPS-referred youth (*n* = 122), official CPS files were analyzed using the MCS. For all participants (including CPS referrals), the Maternal Maltreatment Classification Interview (MMCI; Cicchetti et al., 2003) was conducted with caregivers, videotaped, and coded by trained research assistants. Information from both sources determined severity, timing, and chronicity for each MCS-defined subtype. The MMCI comprises a set of standardized screeners accompanied by follow-up questions to assess the lifetime presence of maltreatment incidents. Coders rated subtype, severity (1 = low to 5 = high), developmental period for each incident (infancy, toddlerhood, preschool age, early school age, late school age, adolescence, early adulthood), and frequency (1 = one-time occurrence; 2 = on less than 50% of days in developmental period; 3 = on more than 50% of days in developmental period). In case of coding issues, a group of senior researchers, chaired by the deputy principal investigator, discussed the case and consulted Dr. Manly if needed. Independent blind raters double-coded 20% of T1 codings, yielding moderate to high inter-rater agreement for maltreatment (CPS file codings: Cohen’s *k* between .58 and .78; caregiver interviews: Cohen’s *k* between .78 and 1.00; Sierau et al., 2017). For CPS-referred youth, information from case records and interviews was pooled at the incident level, using the source with more information on subtype and severity. If CPS records indicated maltreatment but none was reported on the MMCI, ratings were solely based on CPS records. For our hypothesis-driven analyses addressing research questions 1 and 2, we first derived a dichotomous maltreatment status variable indicating the presence of any maltreatment experience across all developmental periods, subtypes, and frequencies. For the exploratory analyses, and based on previous research suggesting differential effects of various forms of maltreatment (e.g., Schlensog-Schuster et al., 2024), we created three variables capturing the presence of abuse (i.e., sexual and/or physical abuse), neglect (i.e., lack of supervision, failure to provide, and/or moral-legal-educational maltreatment), and emotional maltreatment experiences. Additionally, following a cumulative risk approach (e.g., Evans et al., 2013), we summed the number of maltreatment subtypes experienced to reflect differences in the breadth of maltreatment exposure.

To measure caregivers’ maltreatment experiences, we used the German version of the Childhood Trauma Questionnaire (CTQ; Wingenfeld et al., 2010). The CTQ is a valid and reliable (Klinitzke et al., 2012) 28-item questionnaire which consists of 25 clinical items yielding five maltreatment subscales (i.e., sexual abuse, physical abuse, physical neglect, emotional abuse, emotional neglect) and three validity items. For our analyses, we calculated a total score based on individual subscale ratings.

#### Psychiatric Symptoms

For T1, we administered the SDQ (R. Goodman, 1997) to primary caregivers (two, if possible), participants, and teachers to measure participants’ psychiatric symptoms. Previous studies demonstrate that the SDQ is a valid and reliable instrument for assessing psychological symptoms in both clinical and population-based studies (e.g., Becker et al., 2004; Rothenberger et al., 2008). The SDQ consists of 25 items on a three-point scale, yielding five symptom scales (i.e., conduct problems, hyperactivity, emotional problems, peer problems, and prosocial behavior) or two higher-order subscales reflecting internalizing and externalizing symptoms (A. Goodman et al., 2010). In this study, we calculated the two higher-order subscales by averaging the emotional and peer problem subscales (internalizing) as well as the conduct problems and hyperactivity subscales (externalizing) across up to four informants. Lower and higher-order subscales from different informants exhibited good internal consistency (emotional problems: *α* = 0.75, peer problems: *α* = 0.81, conduct problems: *α* = 0.81, hyperactivity: *α* = 0.83, internalizing symptoms: *α* = 0.83, externalizing symptoms: *α* = 0.89). For our analyses, we used the internalizing and externalizing symptom subscale scores.

Additionally, caregiver depression was assessed using the German version of the Patient Health Questionnaire (PHQ-D; Gräfe et al., 2004). The PHQ-D consists of 76 items on two- to four-point scales yielding several symptom subscales (e.g., depression, panic, and eating disorders) and two subscales reflecting psychosocial stressors and functioning. For this study, we exclusively used the caregiver depression subscale score as prior research has demonstrated its exceptionally high validity (Gräfe et al., 2004).

#### Socioeconomic Factors

We assessed several socioeconomic factors via questionnaire for T1. Household net income was measured on a 12-point scale ranging from “less than 500 €” to “more than 5500 €”. Participants’ gender was measured dichotomously (0 = male; 1 = female), while participants’ age was assessed continuously. We also included the number of intermediate study participations (i.e., 0, 1, or 2) based on the respective study documentation as a continuous control variable in the analysis.

#### Appointment Evaluation

We also applied a 5-point Likert scale questionnaire to a subsample to evaluate participants’ and caregivers’ perceived effort of appointment (0 = not at all; 4 = very), relationship with the research assistant (0 = very negative; 4 = very positive), intent to re-participate (0 = definitely not; 4 = definitely), and participants’ enjoyment of the appointment (0 = not at all; 4 = very much). Additionally, research assistants evaluated participants’ and caregivers’ motivation, cooperation, and concentration during the appointment on a 4-point scale (0 = not at all; 3 = very). For our analyses, we calculated a sum score based on individual scores of these three variables to indicate participants’ and caregivers’ overall engagement throughout the appointment.

#### Re-assessment Effort Variables

To measure re-assessment effort for the second wave of data collection, we identified suitable variables in our recruitment documentation file. First, we derived the continuous variable recruitment complexity by counting the number of characters in the comment field of the recruitment documentation, where the research assistant responsible for arranging the appointments recorded information about the family after each contact. Next, we derived the continuous variable recruitment duration in days by calculating the difference between the date of the first contact (attempt) and the last contact before the appointment. We also coded the continuous variable number of appointments, which varied due to cancellations (only cancellations by participants were counted). Furthermore, we coded a continuous variable reflecting the number of communication channels used (e.g., telephone, email, mail, outreach measures). Additionally, we coded the amount of monetary incentivization, which typically averaged 50 € and was adjusted throughout the study for families where we found it necessary, primarily due to increased burden and/or lack of motivation to participate. At the same time, we reduced the monetary incentive for families who were only able to complete questionnaires and could not attend the on-site personal appointments, thereby missing out on interviews and experiments. Moreover, we dichotomously coded (0 = up to date, 1 = not up to date) whether the contact information for families was current and attempted to verify it through the local registration office (address verification attempt). Additionally, we dichotomously coded the success of the address information update (0 = no success; 1 = success, address update success). Finally, we conducted personal home visits to establish contact with families we did not reach otherwise (e.g., telephone, email, letter) and dichotomously coded this (0 = no success; 1 = success, outreach success).

### Data Analysis Plan

Data analysis was carried out in seven steps using R (version 4.3.3; R Core Team, 2021). First, we described attrition rates for the overall sample as well as only for families with whom we were able to establish contact. Second, to test our hypotheses regarding determinants of attrition, we initially ran bivariate analyses (i.e., *χ*^2^- and *t*-tests) with the dependent variable (i.e., attrition) and independent psychosocial and socioeconomic variables (i.e., participants’ and caregivers’ maltreatment status, participants’ and caregivers’ psychopathology, household net income, participant’s age, and gender). Third, as we only assessed participants’, caregivers’, and research assistants’ evaluations of the appointment in a subsample during T1, we explored potential differences between the attrition and retention groups bivariately. Fourth, we conducted logistic regression analyses to examine whether potential bivariate associations between psychosocial and socioeconomic variables and study attrition remained significant after accounting for shared variance and intermediate study participation. To prevent the exclusion of observations, missing values were imputed via predictive mean matching (mice-package; van Buuren & Groothuis-Oudshoorn, 2011) prior to these analyses. Fifth, for the analyses of re-assessment effort differences, we again bivariately (i.e., *χ*^2^-tests, t-tests) analyzed group differences in dependent re-assessment effort variables (i.e., recruitment complexity & duration, number of appointments and communication channels, monetary incentivization, address verification, outreach success) between the maltreated and non-maltreated subsample using the updated information on maltreatment experiences from T2. Sixth, we ran a multivariate analysis of covariance (MANCOVA) with the continuous dependent variables (recruitment complexity & duration, number of appointments and communication channels, monetary incentivization; reduced by those variables only applicable to a subset of the sample such as address verification attempt, address verification success, and outreach success) and participants’ (updated) maltreatment status, controlling for age, gender, as well as caregivers’ household net income. Seventh, as post-hoc analyses, we conducted individual analyses of covariance (ANCOVAs) for each dependent variable to assess which re-assessment effort dimension was most affected by maltreatment experiences, controlling for age, gender, and interim study participation. The *p*-values of the ANCOVAs were Bonferroni-adjusted to correct for multiple testing.

## Results

We were able to reassess *n* = 616 participants from the initial sample (*n* = 863), resulting in an overall attrition rate of 28.6% between T1 and T2. Among those reassessed, *n* = 573 (93.0%) completed the full assessment, including personal appointments with both the participant and the caregiver, as well as questionnaires, interviews, and experiments. In *n* = 15 cases (2.4%), data were collected in person from only one informant (either participant or caregiver), while *n* = 28 (4.5%) completed questionnaires only, either online or via mail. A total of *n* = 745 (86.3%) participants from the initial sample were successfully re-contacted. Of those, *n* = 129 declined to participate, resulting in a contact-specific attrition rate of 17.3% (see Table 2). Unless otherwise stated, all analyses refer to the overall attrition rate across the full sample, regardless of whether contact was re-established.

**Table 2.** Retention and Attrition Overview Between the First (T1) and Second (T2) Data Collection Wave of the AMIS- Sample.

	*N* (%) of total sample	% Of Successfully Re-contacted Participants
Total sample (T1)	863 (100.0%)	-
Successfully Re-Contacted for T2	745 (86.3%)	100.0%
Reassessed (T2)	616 (71.4%)	82.7%
Fully assessed	573 (66.4%)	76.9%
Partially assessed	43 (5.0%)	5.8%
One informant only	15 (1.7%)	2.0%
Online participation only	28 (3.3%)	3.8%
Attrition (T2)	247 (28.6%)	17.3%
Could not be reached for T2	118 (13.7%)	-
Declined to Re-participate	129 (15.0%)	17.3%

### Determinants of Attrition

Bivariate analyses revealed that youth with maltreatment experiences were significantly more likely to drop out of the study, with an odds ratio (OR) of 2.81. Specifically, abuse was associated with a 2.68-fold increase, neglect with a 2.23-fold increase, and emotional maltreatment with a 3.12-fold increase in attrition risk. Furthermore, a higher number of maltreatment subtypes experienced was associated with increased likelihood of leaving the study. The attrition subsample also showed higher levels of caregiver-reported maltreatment histories, more pronounced psychiatric symptoms in both participants and caregivers, lower household net income, older age, male gender, and fewer intermediate study participations (see Table S2). In addition, attrition was associated with lower ratings of relationship quality and intent to re-participate, as reported by participants and caregivers, as well as lower interviewer-rated engagement (see Table S3).

Confirmatory multivariate analyses showed that participants with maltreatment experiences (OR = 1.56), externalizing symptoms (OR = 1.20), lower household net income (OR = 1.14), and fewer intermediate assessments (OR = 4.35) were more likely to drop out (see Table 3). Exploratory multivariate analyses further indicated that emotional maltreatment remained a significant predictor of attrition (OR = 1.62), while abuse and neglect were not. A higher number of maltreatment subtypes was also associated with an increased likelihood of dropout (OR = 1.25, see Tables S4–S5).

**Table 3.** Results of the Logistic Regression Identifying Psychosocial and Socioeconomic Determinants of Study Attrition in the AMIS-Sample.

Variable	B	SE	OR	95% CI	*p*-value
Intercept	−0.186	.47	-	-	.690
Youth’s maltreatment status	0.438	.20	1.55	[1.04, 2.30]	.030
Caregiver’s maltreatment experiences	0.002	.01	1.00	[0.99, 1.01]	.729
Youth’s internalizing symptoms	−0.027	.07	0.97	[0.85, 1.11]	.690
Youth’s externalizing symptoms	0.180	.06	1.20	[1.07, 1.34]	.002
Caregiver’s depression levels	0.012	.02	1.01	[0.97, 1.06]	.569
Household net income	−0.125	.04	0.88	[0.82, 0.95]	.001
Youth’s age	0.037	.03	1.04	[0.98, 1.10]	.189
Youth’s gender	−0.173	.18	0.84	[0.59, 1.19]	.332
Intermediate study participation	−1.460	.16	0.23	[0.17, 0.32]	<.001

*Note.* SE = Standard Error; OR = Odds Ratio; CI = Confidence Interval.

Analyses were conducted using the T1 sample (*n* = 863).

### Differences in Re-Assessment Effort

Results of the bivariate analyses regarding re-assessment effort differences indicated that the recruitment of maltreated participants took longer, involved more appointments and communication channels, and required higher monetary incentivization. Additionally, contact information had to be investigated via the registration office more frequently for the maltreated subsample (see Table S6). Moreover, results of the MANCOVA indicated that maltreated children required more re-assessment effort, considering mean differences in recruitment complexity, recruitment duration, number of appointments, number of communication channels, and monetary incentivization. Furthermore, lower household net income and higher participant age were associated with higher re-assessment effort (see Table 4). Lastly, post-hoc analyses revealed that this global effect was primarily driven by differences in the variables number of communication channels and monetary incentivization (see Table S7).

**Table 4.** Results of the MANCOVA on Differences in Re-Assessment Effort Between Maltreated and Non-maltreated Youth Controlling for Household Net Income, Participant’s Age and Gender, and Interim Study Participation.

Variable	Test statistic	Approx. F	Num df	Den df	*p*-value
(Intercept)	0.43	136.082	5	523	<.001
Youth’s maltreatment status	0.97	3.190	5	523	.008
Household net income	0.95	5.710	5	523	<.001
Youth’s age	0.93	8.349	5	523	<.001
Youth’s gender	0.98	1.757	5	523	.120
Intermediate study participation	0.98	1.602	5	523	.158

*Note.* Analyses were conducted using the T2 sample (*n* = 616).

## Discussion

To our knowledge, this study is the first to examine the determinants of attrition and differences in re-assessment efforts in a large-scale prospective study expressly designed to thoroughly assess the longitudinal effects of maltreatment on children and adolescents. Spanning an average of approximately 8 years between the initial assessment (T1) and a subsequent assessment (T2), 28.6% of T1 participants did not participate in T2. Moreover, both bivariate and multivariate analyses indicate that the presence of maltreatment experiences, externalizing symptoms, household net income, and lack of interim study participation markedly increase the likelihood of attrition. Additionally, re-assessment efforts for the second wave of data collection were substantially higher for the maltreated subsample compared to the non-maltreated subsample.

### Attrition Rates

Compared to other studies in related fields (e.g., Dimakos et al., 2023), our study-specific attrition rate can be considered as relatively low, particularly considering the large time span between data collection waves. Studies with comparably long intervals in similar populations have reported higher attrition rates of 7% annually (Cicchetti & Manly, 1990), for example. At the same time, our attrition rate of 28.6% is slightly higher than the 22% (Negriff et al., 2020) and comparable to the 27% (Kisely et al., 2020) reported in other longitudinal studies with maltreated youth. However, the first three measurement points were closer in time in Negriff et al.’s (2020) research, which may have increased participants’ identification with the study and helped prevent attrition to some extent. Furthermore, the higher contact frequency in this study may have contributed to fewer participants leaving the study due to incorrect contact information in comparison to our study. Additionally, the sample consisted of children aged 9 to 12 years, which is in contrast with our broader age range, potentially resulting in children being more likely to still reside with their caregivers and not having moved away due to pursue vocational degrees, higher education or for other reasons. In light of these considerations, we interpret our attrition rate over the extended time span, especially among families we were able to re-contact, as comparably low as in the study of Negriff et al. (2020). Additionally, it highlights the importance of contact maintenance in longitudinal designs, especially with high-risk populations, which has also been emphasized in studies analyzing effective recruitment strategies (e.g., Dyson et al., 2021).

### Determinants of Attrition

Our findings on the psychosocial and socioeconomic determinants are partially consistent with prior studies employing different samples (Kazdin et al., 1993; Williams et al., 2013). However, multiple factors seem to contribute to this pattern. Our results suggest that a) maltreated youth, b) those with externalizing symptoms, and/or c) those with low economic resources may be at particularly high risk of attrition in longitudinal studies on maltreatment involving children, adolescents, and their caregivers. It is conceivable that limited resources and high stress levels make consistent study participation more difficult for individuals with these characteristics. For example, one might expect that the finding of low household net income would indicate that monetary incentives could serve as an important motivator for economically disadvantaged families to participate in the study. However, our results do not seem to support this assumption, especially considering that we increased the compensation for hard-to-reach families in a flexible manner. Instead, it is more likely that this adjustment enabled us to reassess some economically disadvantaged families who, based on the standard compensation, might not have been able to participate again in the study. This effect, which was evident despite the flexible adjustment of the monetary incentives, may in fact be even stronger than our findings suggest. Therefore, it seems crucial to keep potential barriers to participation in future studies as low as possible to mitigate the systematic exclusion of certain risk groups within at-risk families and minimize the risk of biasing study results. Importantly, while our findings highlight the relevance of these factors, they do not allow conclusions about their potential interplay. Future research could therefore explore whether and how these factors interact in shaping attrition patterns.

In addition, our results underscore the importance of interim study participation and support the interpretation that maintaining a consistently high frequency of contact could be a crucial measure to minimize the risk of study attrition. Higher contact frequency may enhance study identification by, for example, improving recall of study content and recognition of personnel, ultimately increasing the likelihood of participation. However, it remains unclear whether the positive impact of increased contact frequency applies equally across different risk groups. Excessive contact, for instance, could overwhelm or alienate families, particularly those facing multiple psychosocial challenges, thereby deterring further participation. Consequently, we cannot rule out that increased contact frequency benefited easily recruitable families while harder-to-recruit families may still have dropped out. Future studies could address this question through targeted study designs. Nevertheless, we believe it is essential for funding agencies to give stronger consideration to ensuring sufficient and stable resource provision, as this supports robust study infrastructure and effective panel maintenance, both critical for mitigating systematic attrition in longitudinal research with high-risk populations. Moreover, bivariate subsample analyses of appointment evaluations by participants, caregivers, and interviewers highlight the importance of personal contact quality for re-participation. This encompasses not only the connection with research assistants but also minimizing the burden of assessments to maintain high engagement. For instance, in our demanding study, engagement among participants with externalizing symptoms may have been reduced due to lengthy appointment durations, possibly discouraging further participation. Accordingly, we recommend careful consideration of assessment protocol length. Although these data were collected only from a subsample, they provide preliminary insights into the importance of appointment quality in longitudinal studies in this field. Given the significance of re-participation observed here, future studies should examine this topic more thoroughly. Additionally, our findings suggest that incorporating evaluation questionnaires can help identify families at higher risk of attrition due to lower intent to re-participate at an early stage, facilitating the timely implementation of targeted panel maintenance measures.

It is also worth noting that our study found that male gender was associated with a higher attrition rate in bivariate analyses, but this effect was no longer significant when controlling for variables like externalizing symptoms in the multiple regression model. This finding is particularly interesting, as various studies (Chi & Cui, 2020), including our own (e.g., Keil et al., 2019; Schlensog-Schuster et al., 2024), have shown that boys tend to exhibit higher levels of externalizing behaviors. This could suggest that previous studies examining the impact of gender (Delfabbro et al., 2017; Eaton et al., 1992; Kisely et al., 2020) without controlling for externalizing symptoms may have identified gender effects that were at least partly due to higher externalizing symptomatology in boys. However, these interpretations are speculative and warrant further investigation in future studies.

Further investigation into the role of maltreatment experiences reveals a more complex relationship with study attrition, as indicated by our exploratory analyses. On the one hand, our findings indicate that emotional maltreatment, in particular, may function as a driving force for attrition (see Table S4). This aligns with previous research highlighting its strong impact beyond the effects of abuse and neglect (e.g., Schlensog-Schuster et al., 2024). One possible explanation is that families with particularly strained caregiver-child relationships may feel more burdened by appointments or less inclined to confide in interviewers, making them less likely to continue to participate in a study focusing on maltreatment. On the other hand, the null findings for abuse and neglect might be due to lower statistical power, given their lower prevalence in our sample compared to emotional maltreatment. In contrast, the finding that participants with a greater breadth of maltreatment experiences were more likely to drop out (see Table S5) appears to support a cumulative risk perspective (e.g., Evans et al., 2013) on the developmental effects of maltreatment whereby the wear and tear of pervasive levels of adversity may compel individuals to discontinue participation. While these findings are preliminary and require replication, they illustrate the added value of a fine-grained maltreatment assessment, as applied in our study, in capturing the nuanced associations between different forms of maltreatment and study attrition.

### Differences in Re-Assessment Effort

The findings regarding the increased re-assessment effort in families with maltreatment histories are consistent with those of Dyson et al. (2021). This suggests that, especially in comparative research designs, differentiated recruitment strategies could lead to better recruitment success by allocating more resources to high-risk families, potentially offsetting savings made with low-risk families. However, the data from the present study can only provide limited insights into the magnitude of these differences and does not offer a comprehensive picture of the relevant differential associations. Consequently, future studies could systematically explore this question to tailor resource planning more accurately for researchers and funding institutions, while simultaneously minimizing the attrition rate.

Furthermore, our results also demonstrate the influence of socioeconomic factors on re-assessment efforts. In addition to the previously discussed association between low household income and the recruitment process, our results indicate that re-assessing older participants required more effort. This likely reflects challenges typical of the transition from adolescence to young adulthood, such as residential mobility due to education or employment (Delfabbro et al., 2017), and reduced caregiver involvement with increasing age. Nevertheless, this additional effort did not result in higher attrition rates, possibly due to strong familial identification with the study and the effectiveness of directly re-contacting participants, which has been shown to substantially increase re- participation in other studies (Eisner et al., 2018). Moreover, as our sample spanned a broad age range, it is plausible that the relevance of specific predictors varies by age - participant-related factors may play a greater role in older youth, while caregiver characteristics may be more influential in younger children. Supporting this, a meta-analysis identified low parental education, young maternal age, single parenting, and low occupational status as predictors of dropout in parenting programs (Reyno & McGrath, 2006). These findings underscore the importance of collecting contact information from both caregivers and participants early on and highlight the need for future research on age-specific patterns of attrition.

A notable secondary finding is the considerable number of participants for whom maltreatment experiences were not reported at T1, but at T2. This is logical, as the probability of experiencing some form of maltreatment increases with age. However, this finding underscores the value of prospective and repeated measurements of maltreatment and other variables to better capture the dynamic nature of these phenomena. Repeated assessment allows for the documentation and analysis of changes in maltreatment experiences over time, which is essential for understanding long-term impacts and developing effective preventive interventions.

### Practical Observations and Statistical Considerations

In addition to the empirically identified factors, our observations point to further unassessed barriers that likely contributed to systematic attrition. Based on our experience, logistical challenges such as lack of child care for siblings, scheduling conflicts, lengthy assessments, and participant relocation were major obstacles. Although we did not formally evaluate specific retention strategies, a stable research team with consistent, rapport-trained contact persons appeared crucial for long-term engagement. Practical measures - child care support, home visits, phone interviews, and flexible scheduling - seemed to enhance accessibility and support retention. Further strategies, including increased monetary reimbursement and sending birthday or Christmas cards, also appeared helpful. Interestingly, despite mixed findings in the literature (Lindsey et al., 2014), text message reminders proved less effective than expected. Overall, our experiences align with previous findings emphasizing the importance of financial incentives (Graziotti et al., 2012; Jong et al., 2023), practical supports (Lindsey et al., 2014), and rapport-building strategies such as home visits and flexible scheduling (Graziotti et al., 2012).

While retention efforts are vital, systematic attrition remains a major challenge in longitudinal research, potentially biasing statistical estimates if unaddressed (Dimakos et al., 2023; Ellard-Gray et al., 2015; Huque et al., 2018). Various methods exist to mitigate such bias, including the use of sample weights that adjust for differential retention probabilities (Seaman & White, 2013). By assigning weights inversely proportional to participation probability, underrepresented groups can be up weighted to restore representativeness. However, the method must suit the scope and design of the study, as not all systematic attrition necessarily biases the estimate. For example, in (longitudinal) randomized controlled trials, attrition linked to variables like maltreatment status may distort treatment effect estimates. In these cases, approaches such as pattern-mixture models (Little, 1993), G-computation (Snowden et al., 2011), and inverse probability of treatment weighting (IPTW; Cole & Hernán, 2008) can reduce bias. IPTW, which reweights observed cases to approximate the original sample, is commonly implemented via logistic regression - as applied in the present study - under the assumption that attrition is driven by observed covariates. Though randomization in RCTs reduces unmeasured confounding, bias may still arise if attrition relates to unobserved effect modifiers.

### Limitations

Several limitations of this study warrant consideration. While the findings provide valuable insights into attrition and re-assessment efforts in longitudinal research with maltreated and non-maltreated youth, they should be generalized with caution. First, the recruitment methods may have introduced sampling bias. Specifically, relying on child protection and psychiatric services might have disproportionately included participants from certain socio-economic backgrounds or those with specific maltreatment experiences, potentially limiting the representativeness of the sample. Second, the psychosocial burden of the COVID-19 pandemic likely added an additional challenge to recruitment, particularly for high-risk families. Although strategies such as home visits were implemented to address this issue, and the attrition rate is interpreted as relatively low in this context, selection bias due to the pandemic cannot be entirely ruled out. Third, the use of self-report measures to assess maltreatment experiences, psychopathology, and socio-economic factors introduces the risk of response bias. Participants may have under-reported sensitive information due to social desirability or memory biases, leading to incomplete or distorted data. Additionally, maintaining consistency across multiple waves of data collection in longitudinal studies presents inherent challenges. Variations in research personnel, measurement tools, or study protocols over time may have introduced inconsistencies, potentially affecting the reliability and validity of the findings. Furthermore, unmeasured variables such as significant life events, changes in family circumstances, or participant motivation may have influenced attrition but were not accounted for in this study. The study’s geographic focus presents another limitation. Conducted in specific locations, it may not fully capture the diversity of experiences across different cultural or socio-economic contexts. Replication studies in more diverse populations are needed to validate the findings. Lastly, the broad age range of participants may limit the generalizability to populations with narrower or different age groups. Attrition rates and their determinants may vary across developmental stages. Similarly, the relatively long intervals between measurement points could have influenced attrition differently compared to studies with shorter intervals, potentially obscuring short-term dynamics. Future research should consider these temporal aspects to better understand how they impact attrition.

### Conclusion

In conclusion, this study sheds light on the determinants of attrition and differences in re-assessment efforts in longitudinal studies involving maltreated and non-maltreated children and adolescents. The findings emphasize the importance of maintaining contact, particularly with high-risk populations, to minimize attrition rates. Moreover, the study highlights the need for tailored recruitment strategies, such as allocating more resources to recruit high-risk families, to enhance participation rates and reduce bias in study findings. Future research could benefit from person-centered data analysis methods to identify vulnerable subgroups prone to attrition, thereby informing targeted retention efforts and improving the overall robustness of longitudinal studies in this field. Understanding the factors influencing attrition and re-assessment efforts has broader implications for informing intervention strategies and policy decisions. Additionally, by identifying and addressing barriers to participant engagement, researchers can enhance the effectiveness of interventions targeting vulnerable populations and contribute to evidence-based policy initiatives aimed at supporting at-risk youth and families. However, further research is needed to deepen our understanding of the complex interplay between participant characteristics, study design factors, and attrition rates in longitudinal research with maltreated youth. Longitudinal studies employing innovative methodologies, such as advanced statistical techniques or mixed-methods approaches, could provide more nuanced insights into attrition mechanisms and facilitate the development of tailored retention strategies.

## Supplemental Material

Supplemental Material - Lost Narratives: Identifying Predictors of Attrition and Differences in Recruitment Effort in a Longitudinal Study on Child Maltreatment Supplemental Material for Lost Narratives: Identifying Predictors of Attrition and Differences in Recruitment Effort in a Longitudinal Study on Child Maltreatment by Jan Keil, Josephine Breuer, Romy Küchler, Angelika J. Bracher, Charlotte C. Schulz, Dorukhan Açıl, Sarah Bergmann, Nina Alexander, Tobias Stalder, Robert Miller, Maria Licata-Dandel, Volker Mall, Michaela Augustin, Anne Sophie Wenzel, Georg von Polier, Daniel Radeloff, Kai von Klitzing, & Lars O. White in Child Maltreatment

## ORCID iDs

Jan Keil https://orcid.org/0000-0002-5001-0659

Angelika J. Bracher https://orcid.org/0000-0002-3989-7244

Charlotte C. Schulz https://orcid.org/0000-0002-1082-4877

Dorukhan Açıl https://orcid.org/0009-0005-4986-4154

Sarah Bergmann https://orcid.org/0009-0006-4909-1996

Robert Miller https://orcid.org/0000-0002-8665-5248

Maria Licata-Dandel https://orcid.org/0000-0001-9898-0489

Michaela Augustin https://orcid.org/0000-0003-3279-2316

Anne Sophie Wenzel https://orcid.org/0009-0003-2698-460X

Daniel Radeloff https://orcid.org/0000-0002-8430-8306

Kai von Klitzing https://orcid.org/0000-0003-3010-5121

Lars O. White https://orcid.org/0000-0001-7185-9929
