# Supplementary material for: Lost Narratives: Identifying Predictors of Attrition and Differences in Recruitment Effort in a Longitudinal Study on Child Maltreatment
Source: Child Maltreat. 2025 Jul 4;31(2):387–99. doi: 10.1177/10775595251352425 (PMC12992637; doi:10.1177/10775595251352425)
Supplement: Supplemental Material - Lost Narratives: Identifying Predictors of Attrition and Differences in Recruitment Effort in a Longitudinal Study on Child Maltreatment [file sj-pdf-1-cmx-10.1177_10775595251352425.pdf]

Supplementary Information

Table S1

Overview of Data Collection Waves and Assessment Characteristics in the AMIS- Study.

**Timepoint**	**Data collection wave 1 (T1)**	**Interim assessment 1**	**Interim assessment 2**	**Data collection wave 2 (T2)**
Time period	2012-2015	2016-2017	2018-2020	2019-2023
Study site	Munich, Leipzig	Leipzig	Leipzig	Leipzig
Sample size	*N*=966 (*n*=863 in Leipzig; *n*=103 Munich)	*n*=636	*n*=110	*N*=616
Informants	Participants, primary and secondary caregivers, teachers	Participants, primary caregivers	Participants, primary caregivers	Participants, primary and secondary caregivers, teachers
Assessment type	In person	Remote (mail/web-based)	In person	In person
Measures	Interviews, questionnaires, experimental tasks, biological samples	Questionnaires	Interviews, questionnaires, experimental tasks, biological samples, fMRI, rsMRI	Interviews, questionnaires, experimental tasks, biological samples

Note. fMRI = functional magnetic resonance imaging; rsMRI = resting-state magnetic resonance imaging.

Table S2

Results of the bivariate analysis on maltreatment, psychiatric symptom and socioeconomic variable differences between retention and attrition subsamples of the AMIS-study.

**Variable**	**Overall** (n = 863)	**Retention** (n = 616)	**Attrition** (n = 247)	**Test Statistic**	***p*-value**
Youth's Maltreatment Status (% yes)	317 (36.7%)	183 (29.7%)	134 (54.3%)	$\chi^2(1) = 45.356$	<.001
Youth's Abuse Status (% yes)	101 (12.0%)	53 (8.7%)	48 (20.4%)	$\chi^2(1) = 20.852$	<.001
Youth's Neglect Status (% yes)	186 (22.1%)	109 (18.0%)	77 (32.8%)	$\chi^2(1) = 20.735$	<.001
Youth's Emotional Maltreatment Status (% yes)	227 (27.0%)	123 (20.3%)	104 (44.3%)	$\chi^2(1) = 48.310$	<.001
Youth's Number of Maltreatment Subtypes (*M*, *SD*)	0.84 (1.35)	0.60 (1.11)	1.44 (1.68)	$t(840) = 8.464$	<.001
Caregiver's Maltreatment Exposure (*M*, *SD*)	14.12 (16.54)	12.42 (15.03)	18.75 (19.38)	$t(762) = 4.787$	<.001
Youth's Internalizing Symptoms (*M*, *SD*)	2.29 (1.52)	2.12 (1.48)	2.73 (1.53)	$t(862) = 5.281$	<.001
Youth's Externalizing Symptoms (*M*, *SD*)	2.94 (1.86)	2.60 (1.71)	3.78 (1.96)	$t(862) = 8.639$	<.001
Caregiver's Depression Levels (*M*, *SD*)	4.71 (4.26)	4.43 (4.10)	5.45 (4.59)	$t(833) = 3.125$	.002
Household Net Income (Median)	2000–2500€	2500–3000€	1500–2000€	$z = –7.781$	<.001
Youth's Age at T1 (*M*, *SD*)	10.23 (3.17)	10.10 (3.15)	10.55 (3.19)	$t(862) = 1.994$	.058
Youth's Gender (female, % yes)	411 (47.6%)	308 (50.0%)	103 (41.5%)	$\chi^2(1) = 4.749$	.025
Interim Study Participation (*M*, *SD*)	0.86 (0.59)	1.01 (0.53)	0.51 (0.58)	$t(862) = –12.065$	<.001

Note. Analyses were conducted using the T1 sample (N = 863).

Table S3

Results of the t-test analysis of Youth's, Caregivers's, and Interviewer's appointment evaluations by retention and attrition groups.

**Variable**	**N**	**Overall** (*Mean*)	**Retention** (*Mean*)	**Attrition**	**Test Statistic**	***p*-value**	**Bonf.-adj. *p*-value**
Youth’s Effort of Appointment	431	0.63	0.62	0.66	$t(429) = 0.456$	.649	.973
Youth’s Relationship with Interviewer	430	3.82	3.86	3.72	$t(428) = -2.942$	.003	.031
Youth’s Intent to Re-participate	430	3.54	3.61	3.34	$t(428) = -3.187$	.002	.014
Youth’s Enjoyment of Appointment	429	3.48	3.50	3.42	$t(427) = -1.092$	.276	.825
Caregiver’s Effort of Appointment	442	0.61	0.58	0.69	$t(440) = 1.325$	.186	.654
Caregiver’s Relationship with Interviewer	442	3.85	3.88	3.75	$t(440) = -3.252$	.001	.011
Caregiver’s Intent to Re-participate	440	3.61	3.66	3.47	$t(438) = -2.930$	.004	.032
Youth’s Engagement (Interviewer)	448	2.75	2.77	2.68	$t(446) = -2.183$	.030	.178
Caregiver’s Engagement (Interviewer)	450	2.47	2.50	2.38	$t(448) = -2.382$	.018	.123

Note. Bonf.-adj. = Bonferroni-adjusted. Analyses were conducted using a subsample of the T1 sample.

Table S4

Results of the exploratory logistic regression analyzing different types of maltreatment exposure (i.e., abuse, neglect and emotional maltreatment), as well as psychosocial and socioeconomic factors, as determinants of study attrition in the AMIS- sample.

Variable	B	SE	OR	95% CI	*p*-value
(Intercept)	-0.125	.47	-	-	.793
Youth’s abuse status	0.186	.28	1.20	[0.69, 2.10]	.513
Youth’s neglect status	-0.097	.23	0.91	[1.06, 2.36]	.670
Youth’s emotional maltreatment status	0.486	.22	1.63	[1.06, 2.36]	.030
Caregiver’s Maltreatment Experiences	0.002	.01	1.00	[0.99, 1.01]	.783
Youth’s Internalizing Symptoms	-0.025	.07	0.98	[0.86, 1.12]	.708
Youth’s Externalizing Symptoms	0.181	.06	1.20	[1.08, 1.35]	.002
Caregiver’s Depression Levels	0.011	.02	1.01	[0.97, 1.06]	.626
Household Net Income	-0.122	.04	0.89	[0.83, 0.96]	.001
Youth’s Age	0.034	.03	1.03	[0.98, 1.09]	.231
Youth’s Gender	-0.193	.18	0.82	[0.61, 1.23]	.282
Intermediate Study Participation	-1.467	.16	0.23	[0.16, 0.31]	<.001

Note. SE = Standard Error; OR = Odds Ratio; CI = Confidence Interval. Analyses were conducted using the T1 sample (*N* = 863).

Table S5

Results of the exploratory logistic regression analyzing different types of maltreatment exposure (i.e., abuse, neglect and emotional maltreatment), as well as psychosocial and socioeconomic factors, as determinants of study attrition in the AMIS- sample.

**Variable**	**B**	**SE**	**OR**	**95% CI**	***p*-value**
(Intercept)	-0.122	.46	-	-	.791
Youth’s Nr. of Maltreatment Subtypes	0.220	.07	1.25	[1.08, 1.44]	.003
Caregiver’s Maltreatment Experiences	0.000	.01	1.00	[0.99, 1.01]	.929
Youth’s Internalizing Symptoms	-0.021	.07	0.98	[0.86, 1.12]	.762
Youth’s Externalizing Symptoms	0.156	.06	1.17	[1.04, 1.31]	.009
Caregiver’s Depression Levels	0.010	.02	1.01	[0.97, 1.05]	.633
Household Net Income	-0.120	.04	0.89	[0.82, 0.95]	.001
Youth’s Age	0.038	.03	1.03	[0.98, 1.10]	.173
Youth’s Gender	-0.200	.18	0.82	[0.57, 1.16]	.265
Intermediate Study Participation	-1.478	.16	0.23	[0.16, 0.31]	<.001

Note. SE = Standard Error; OR = Odds Ratio; CI = Confidence Interval. Analyses were conducted using the T1 sample (*N* = 863).

Table S6

Results of the bivariate analysis on overall re-assessment effort as well as differences between the non-maltreated and maltreated subsamples of the AMIS-study.

**Variables**	**Overall**	**Non-maltreated**	**Maltreated**	**Coefficients**	***p*-value**
Recruitment Complexity (*M*, *SD*)	877.7 (679.3)	832.0 (530.7)	921.7 (794.9)	*t*(614)=1.64	.102
Recruitment Duration (*M*, *SD*)	141.6 (208.8)	120.6 (173.7)	161.8 (236.3)	*t*(613)=2.46	.014
Nr. of appointments (*M*, *SD*)	1.5 (0.9)	1.4 (0.8)	1.5 (1.0)	*t*(614)=1.01	.314
Nr. of communication channels (*M*, *SD*)	1.4 (1.0)	1.2 (0.7)	1.6 (1.2)	*t*(614)=4.44	<.001
Reimbursement (*M*, *SD*)	49.1 (10.9)	47.5 (7.8)	50.6 (13.2)	*t*(612)=3.43	.001
Address verification attempt (*M*, *SD*)	52 (8.4)	11 (21.2)	41 (78.8)	χ2(1)=16.46	<.001
Address update success (*M*, *SD*)	35 (68.6)	4 (11.4)	31 (88.6)	χ2(1)=3.23	.073
Reach out success (*M*, *SD*)	39 (27.3)	7 (17.9)	32 (82.1)	χ2(1)=1.48	.224

Note. Analyses were conducted using the T2 sample (*n* = 616).

Table S7

Results of the post-hoc individual ANOVAs and Bonferroni-adjusted p-values on differences in re-assessment complexity, re-assessment duration, nr. of appointments, nr. of communication channels and monetary incentivation between maltreated and non-maltreated youth.

**Variables**	**Df**	**F**	***p*-value**	**Bonf.-adj. *p*-value**
Re-assessment complexity	1	1.24	.265	.999
Re-assessment duration	1	4.88	.028	.140
Nr. of appointments	1	0.97	.324	.999
Nr. of communication channels	1	17.83	<.000	<.000
Monetary incentivation	1	9.24	.002	.010

Note. Bonf.-adj. = Bonferroni-adjusted. Analyses were conducted using the T2 sample (n = 616).
